# *NnWOX1-1*, *NnWOX4-3*, and *NnWOX5-1* of lotus (*Nelumbo nucifera* Gaertn)promote root formation and enhance stress tolerance in transgenic *Arabidopsis thaliana*

**DOI:** 10.1186/s12864-023-09772-w

**Published:** 2023-11-28

**Authors:** Liu quan, Liang Shiting, Zhao Chen, Han Yuyan, Zhao Minrong, Li Shuyan, Cheng Libao

**Affiliations:** 1 College of Horticulture and landscape Architechture, Yangzhou University, Jiangsu, People’s Republic of China; 2 College of Guangling, Yangzhou University, Jiangsu, People’s Republic of China

**Keywords:** Lotus, Adventitious root, *NnWOX1-1*, *NnWOX4-3*, *NnWOX5-1*, *Arabidopsis*

## Abstract

**Background:**

Adventitious roots (ARs) represent an important organ system for water and nutrient uptake in lotus plants because of degeneration of the principal root. The *WUSCHEL-related homeobox* (*WOX*) gene regulates plant development and growth by affecting the expression of several other genes. In this study, three *WOX* genes, *NnWOX1-1*, *NnWOX4-3*, and *NnWOX5-1*, were isolated and their functions were assessed in *Arabidopsis* plants.

**Results:**

The full lengths of *NnWOX1-1*, *NnWOX4-3*, and *NnWOX5-1* were 1038, 645, and 558 bp, encoding 362, 214, and 185 amino acid residues, respectively. Phylogenetic analysis classified *NnWOX1-1* and *NnWOX4-3* encoding proteins into one group, and *NnWOX5-1* and *MnWOX5* encoding proteins exhibited strong genetic relationships. The three genes were induced by sucrose and indoleacetic acid (IAA) and exhibited organ-specific expression characteristics. In addition to improving root growth and salt tolerance, *NnWOX1-1* and *NnWOX4-3* promoted stem development in transgenic *Arabidopsis* plants. A total of 751, 594, and 541 genes, including 19, 19, and 13 respective genes related to ethylene and IAA metabolism and responses, were enhanced in *NnWOX1-1*, *NnWOX4-3*, and *NnWOX5-1* transgenic plants, respectively. Further analysis showed that ethylene production rates in transgenic plants increased, whereas IAA, peroxidase, and lignin content did not significantly change. Exogenous application of ethephon on lotus seedlings promoted AR formation and dramatically increased the fresh and dry weights of the plants.

**Conclusions:**

*NnWOX1-1*, *NnWOX4-3*, and *NnWOX5-1* influence root formation, stem development, and stress adaptation in transgenic *Arabidopsis* plants by affecting the transcription of multiple genes. Among these, changes in gene expression involving ethylene metabolism and responses likely critically affect the development of *Arabidopsis* plants. In addition, ethylene may represent an important factor affecting AR formation in lotus seedlings.

**Supplementary Information:**

The online version contains supplementary material available at 10.1186/s12864-023-09772-w.

## Background

Lotus is a member of the Nymphaeaceae family [1, 2] and contains three subgroups (lotus root, seed lotus, and flowering lotus) according to usage. Lotus root is an essential vegetable in southern China [3], the region with the highest cultivation area of the aquatic vegetable within the country. Lotus root originated in China and its diverse products include lotus tea, lotus drinks, glutinous rice lotus, and salt lotus. These products are exported to Japan, South Korea, Europe, and the United States, resulting in substantial economic benefits for local farmers. Therefore, the planting area of lotus root has increased in open-air or protected facilities. In addition, lotus root can be used as an ingredient in Chinese herbal medicine [4], which is beneficial for human health. Generally, the storage organ is formed underground, and adventitious roots (ARs) are necessary for water and nutrient uptake owing to degeneration of the principal root. The development of ARs directly affects the yield and quality of lotus.

The ARs, usually derived from the root primordia, are always located in the hypocotyl, stem, and leaf, and strongly influence plant fixation as well as water and nutrient absorption [5, 6]. Therefore, ARs play an important role in plant growth, especially in those plants where the principal root has degenerated. Plant ARs are classified as a secondary root system, and three biological developmental processes that include the induction, initiation, and expression stages are observed during AR formation [7, 8]. In the first biological process, there is a transition of cell function, and undifferentiated cells develop into meristematic cells, which can further develop into ARs. The secondary biological stage is the primordial-established stage, and meristematic cells differentiate into primordial ARs [9]. In the third stage, the primordial ARs continually develop and break through the stem or leaf epidermis [10].

The formation of ARs is strictly regulated by genetic and environmental factors [11, 12]. Plant hormones are involved throughout the developmental stages of root formation [13]. Ethylene and indoleacetic acid (IAA) are considered the “promoters” of these three biological stages [14, 15]. In rice, ethylene affects AR formation by inducing the death of epidermal cells at the site of AR emergence [14]. Tomato seedlings treated with 1-aminocyclopropane-1-carboxylic acid (ACC, an intermediate metabolite in ethylene synthesis) contain more ARs than control plants [16]. IAA is also a critical factor that regulates AR formation in the plant kingdom [17]. Increasing the endogenous IAA content induces cell differentiation to form ARs [18], and anything that affects IAA synthesis and transport can alter the development of the lateral roots [19, 20]. The crosstalk between ethylene and IAA in regulating root formation shows that the AR formation process is highly complex and many physiological and biological metabolic processes are involved [21]. The formation of lotus ARs occurs in the hypocotyls of seedlings and the internodes of rhizomes [22]. Like in other plants, the formation of ARs in lotus is regulated by ethylene and IAA. Low concentrations of IAA promote cell differentiation, whereas high concentrations of exogenous IAA have the inverse effect [23]. Light and photosynthetic products also greatly influence AR formation [24, 25]. Lignin is closely related to AR formation in lotus seedlings. Increasing lignin content by overexpressing *NnLAC17* leads to a decrease in root number in transgenic *Arabidopsis* plants [26], suggesting that the development of ARs in lotus plants involves a complex regulatory network. Additionally, abscisic acid (ABA) and cytokinins are involved in AR formation [27]. Gene regulation is a genetic factor that affects AR formation. Therefore, understanding the characteristics of gene expression is an effective method to monitor AR development. Several genes participate in AR development. The *puroindoline* (*PIN*) and *AUX* genes encode proteins that are influx and efflux carriers for IAA transport, which influence lateral root formation [28]. *PIN* is highly expressed in primordial organs [29]. Some genes with a lateral organ boundaries (LOB) domain are induced by ethylene and IAA and their expression is necessary for root primordium development. For example, the protein encoded by *ADP-ribosylation factor-like GTPase 1* (*ARL1*), with its LOB domain, strongly influences cell dedifferentiation at the induction stage of ARs [30].

The *WUSCHEL-related homeobox* (*WOX*) gene family consists of several homeodomain transcription factors [31]. The members of the *WOX* family can be classified into three clades: ancient, intermediate, and *WUSCHEL* (*WUS*) [32–34]. This provides evidence that *WOX* influences stress adaptation and root formation in plants [35–38]. Some members of the *WOX* family regulate root development in the initial stage, which is triggered by auxins [39–41]. A similar role was found for *WOX11*, which regulates root architecture in *Arabidopsis* [42]. The overexpression of poplar *WOX5a* can compensate for a loss of function mutation in *WOX5* in the *Arabidopsis* endogen [43]. Liu et al. [44] observed that several *Populus tomentosa WOXs* have different expression profiles, and their functions are the same for AR regeneration, thereby suggesting that WOXs are required for AR development.

In a previous study, we discovered that sucrose and IAA affected AR formation in lotus seedlings. At the same time, we also found that there was a crosstalk between the IAA and ethylene signaling pathways during AR formation [23, 25]. Based on transcriptome data, three transcription factors, *NnWOX1-1*, *NnWOX4-3*, and *NnWOX5-1*, were upregulated after IAA and sucrose treatment. Therefore, we assumed there were close connections between these three genes and hormones during AR development. In the present study, these three genes were cloned, and their expression profiles and functions were analyzed to further confirm the roles of these genes in AR development. In addition, a possible regulatory pathway of *NnWOX1-1*, *NnWOX4-3*, and *NnWOX5-1* was explored.

## Results

### Cloning of *NnWOX1-1*, *NnWOX4-3*, and *NnWOX5-1*

**Fig. 1:**
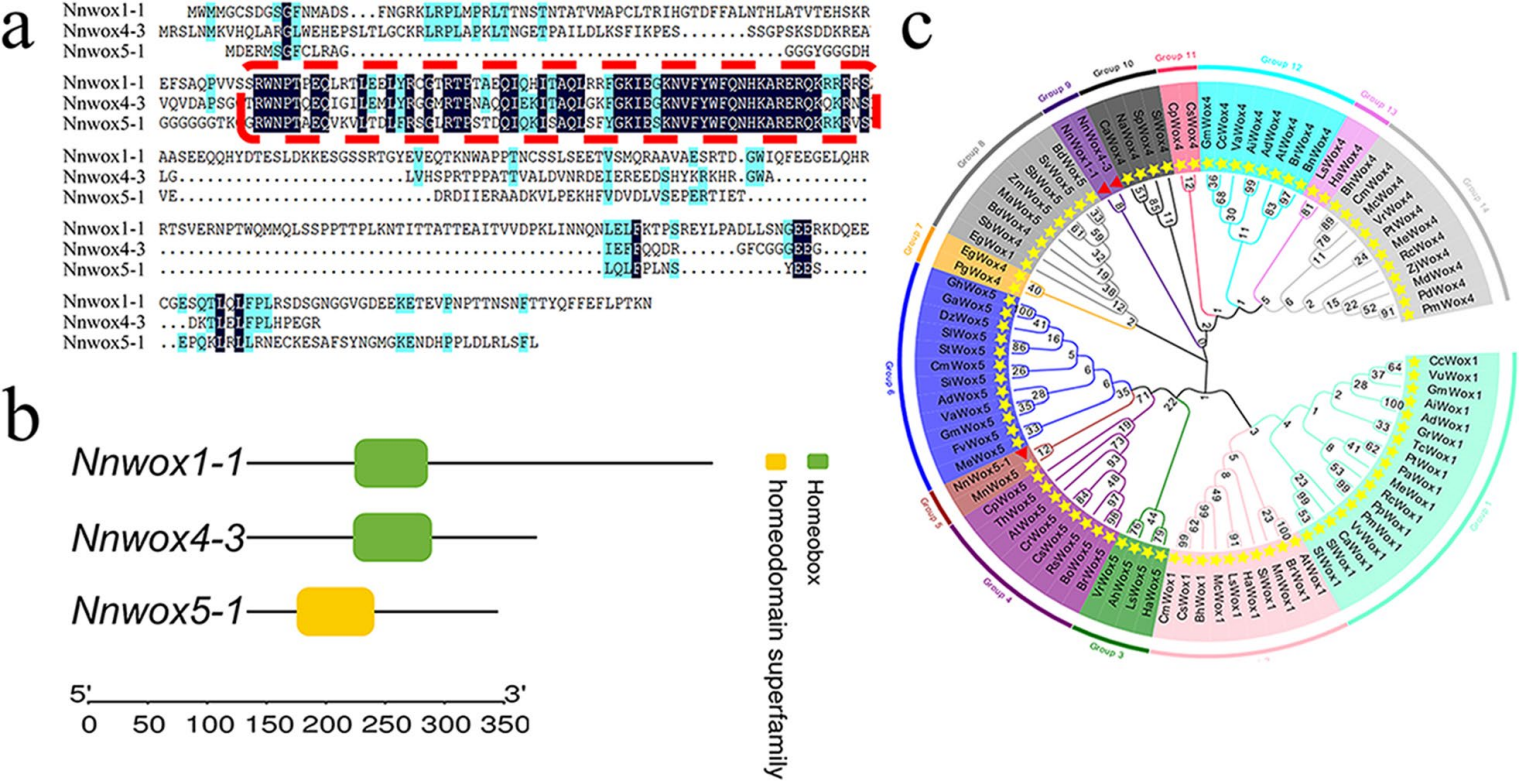
Comparison and phylogenetic tree analysis of Nnwox1-1, Nnwox4-3, and Nnwox5-1. **a** Comparison of *NnWOX1-1, NnWOX4-3*, and *NnWOX5-1* with amino acid sequences. The red box represents the homologous region of the three genes. **b** Domain analysis of *NnWOX1-1-*, *NnWOX4-3-*, and *NnWOX5-1-*encoded proteins, and boxes of different colors represent conserved regions. **c** Phylogenetic tree analysis of *NnWOX1-1-*, *NnWOX4-3-*, and *NnWOX5-1*-encoded proteins with *WOX*-encoded proteins of other species. Fourteen groups with different colors were detected. The red triangle represents the positions of the three proteins in the phylogenetic tree

**Fig. 2:**
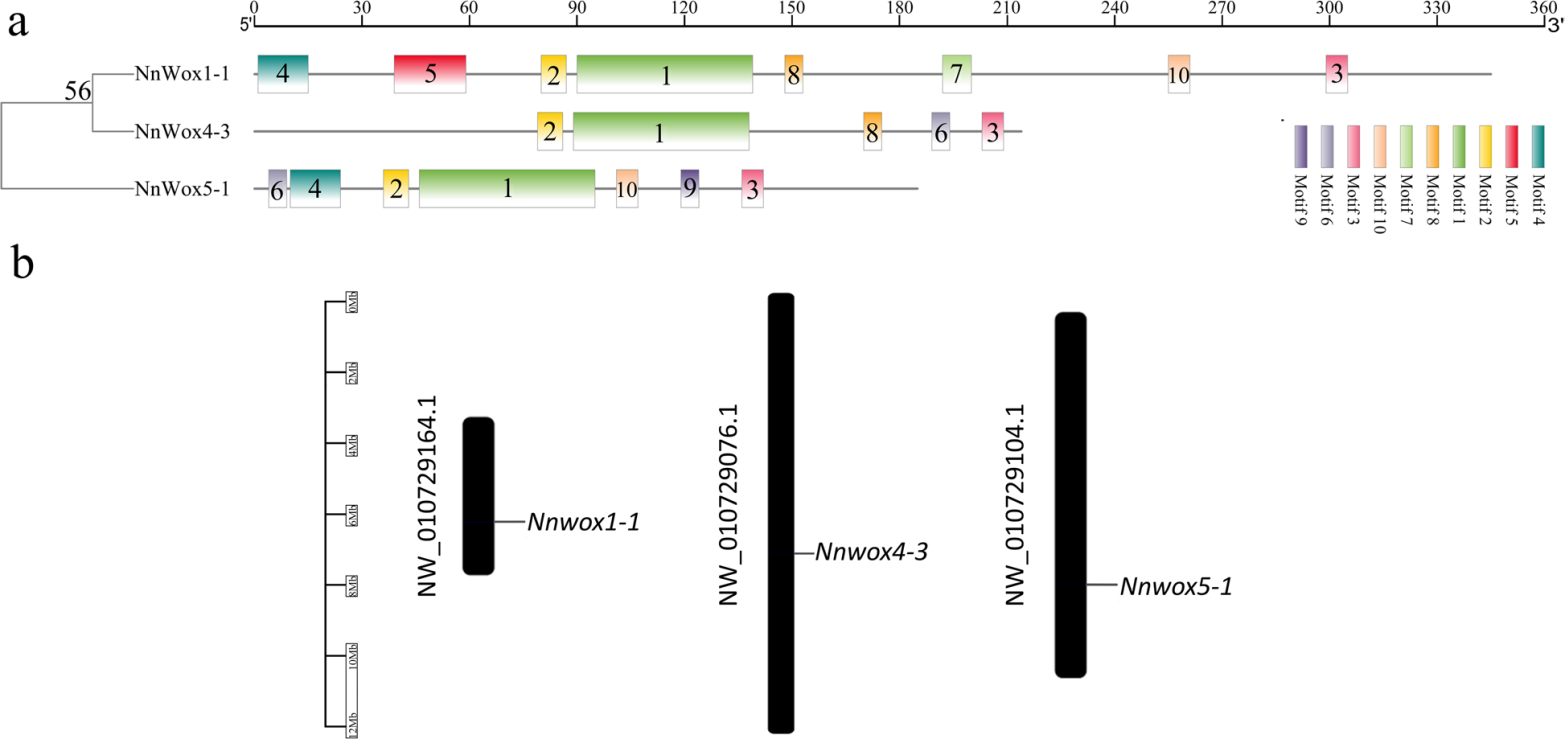
Conserved motif and chromosomal localization analysis of *NnWOX1-1-*, *NnWOX4-3-*, and *NnWOX5-1*-encoded proteins in the whole lotus genome. **a** Distribution of conserved motifs in *NnWOX1-1-*, *NnWOX4-3-*, and *NnWOX5-1*-encoded proteins. Boxes of different colors represent the ten putative motifs, and the boxes with the same color represent the same motif in the structure of these three genes. **b** Chromosomal localization analysis of *NnWOX1-1*, *NnWOX4-3*, and *NnWOX5-1*

Three *WOXs* were cloned via reverse transcription-polymerase chain reaction (RT-PCR). We observed that the full lengths of these three genes were 1038, 645, and 558 bp and encoded 345, 214, and 186 amino acid residues, respectively (Additional Table 1). After comparing with data from the National Center for Biotechnology Information (NCBI) database, it was observed that the three genes contained domains that are conserved in the *WOX* family from *Arabidopsis*, rice, corn, and peanut (Additional Fig. 1). Therefore, these genes were designated as *NnWOX1-1, NnWOX4-3*, and *NnWOX5-1*. The proteins encoded by *NnWOX1-1*, *NnWOX4-3*, and *NnWOX5-1* had conserved regions, although little homology was found in these sequences (Fig. 1a). Further analysis revealed that the *NnWOX1-1-* and *NnWOX4-3-*encoded proteins each contained a homeobox domain, whereas the *NnWOX5-1*-encoded protein contained a superfamily homeodomain (Fig. 1b). The *WOX1-*, *WOX4-*, and *WOX5*-encoded proteins were subdivided into 14 groups. The *NnWOX1-1-* and *NnWOX4-3-*encoded proteins displayed distant relationships with others species and were classified into a single subgroup. The *NnWOX5-1-*encoded protein was closely related to that of *MnWOX5* and was classified into another subgroup (Fig. 1c). Ten motifs were identified in the *NnWOX1-1-*, *NnWOX4-3-*, and *NnWOX5-1*-encoded proteins, which contained eight, five, and seven motifs, respectively. Motifs 1, 2, and 3 were observed in all three proteins, whereas motifs 5 and 7 were observed only in the *NnWOX1-1*-encoded protein (Fig. 2a). Chromosomal localization was also performed; *NnWOX1-1*, *NnWOX4-3*, and *NnWOX5-1* were located at NW-010729164.1, NW-010729076.1, and NW-010729104.1, respectively (Fig. 2b).

### Expression analysis of *NnWOX1-1, NnWOX4-3*, and *NnWOX5-1*

**Fig. 3:**
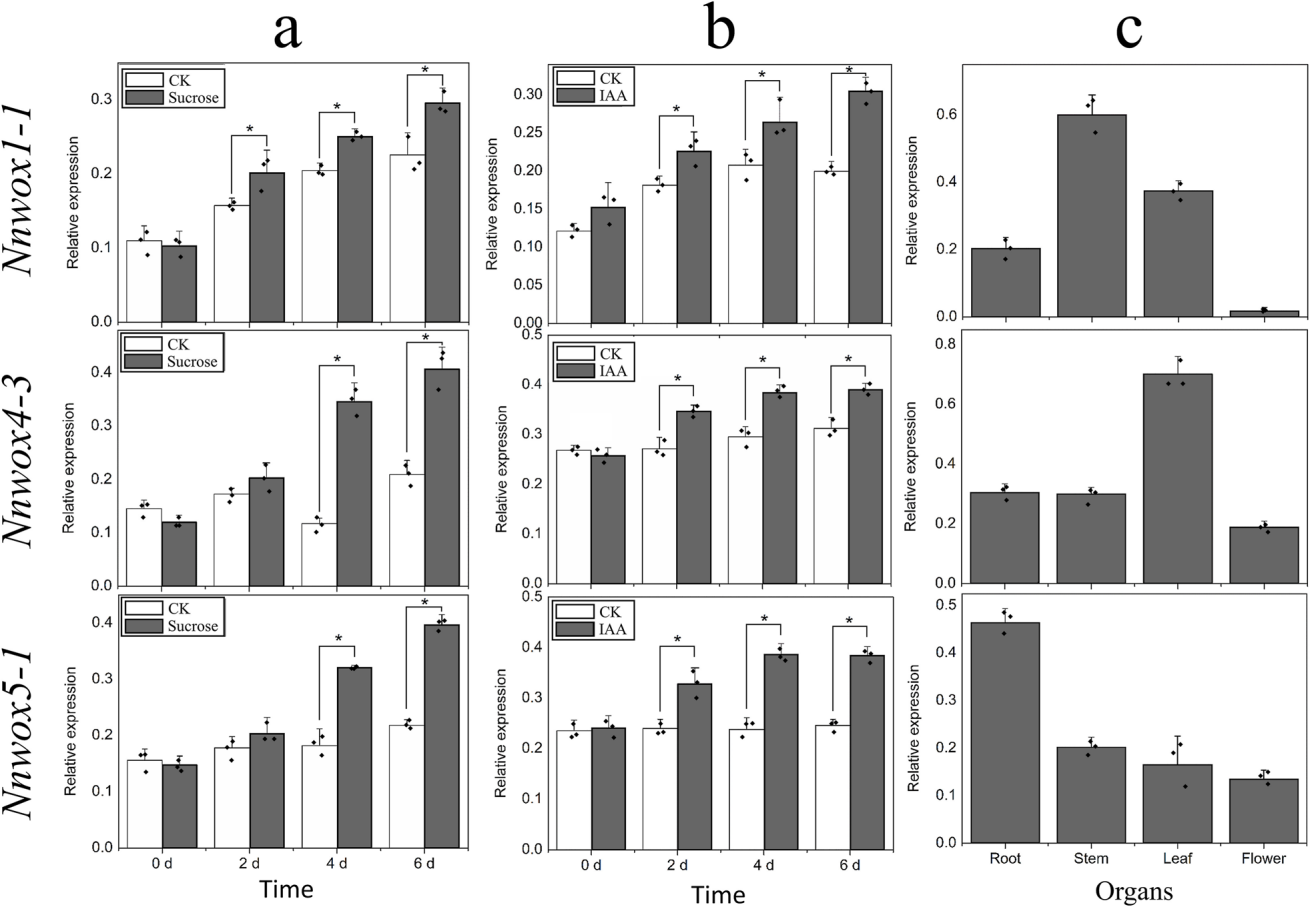
Expression patterns of *NnWOX1-1*, *NnWOX4-3*, and *NnWOX5-1* with different treatments and in different organs, as determined by RT-qPCR. **a** Expression analysis after sucrose treatment. **b** Identification of gene expression in lotus seedlings treated with IAA. **c** Organ-specific expression analysis in lotus plant roots, stems, leaves, and flowers. The mean expression values were calculated from three independent biological replicates, and “*” indicates values that are significantly different compared with control plants (*P* < 0.05)

Reverse transcription quantitative PCR (RT-qPCR) was used to monitor the expression profiles of *NnWOX1-1*, *NnWOX4-3*, and *NnWOX5-1* in lotus seedlings treated with sucrose and IAA. The expression of these three genes increased in response to sucrose treatment. *NnWOX1-1* exhibited an enhanced mRNA level after 2 d, and *NnWOX4-3* and *NnWOX5-1* expression increased after 4 d of treatment. The same expression pattern was observed in response to IAA treatment. The transcriptional levels of *NnWOX1-1*, *NnWOX4-3*, and *NnWOX5-1* were enhanced 2 d after IAA treatment and exhibited an increasing tendency within 6 d. Tissue-specific expression analysis showed that these three genes had different expression patterns in different organs. *NnWOX1-1* mRNA levels were higher in the stem than in the ARs, leaves, and flowers. Increased *NnWOX4-3* expression was observed in leaves, although the gene was expressed in other organs. The transcriptional level of *NnWOX5-1* was higher in ARs than in leaves, stems, and flowers (Fig. 3).

### Functional analysis of genes for root development

**Fig. 4:**
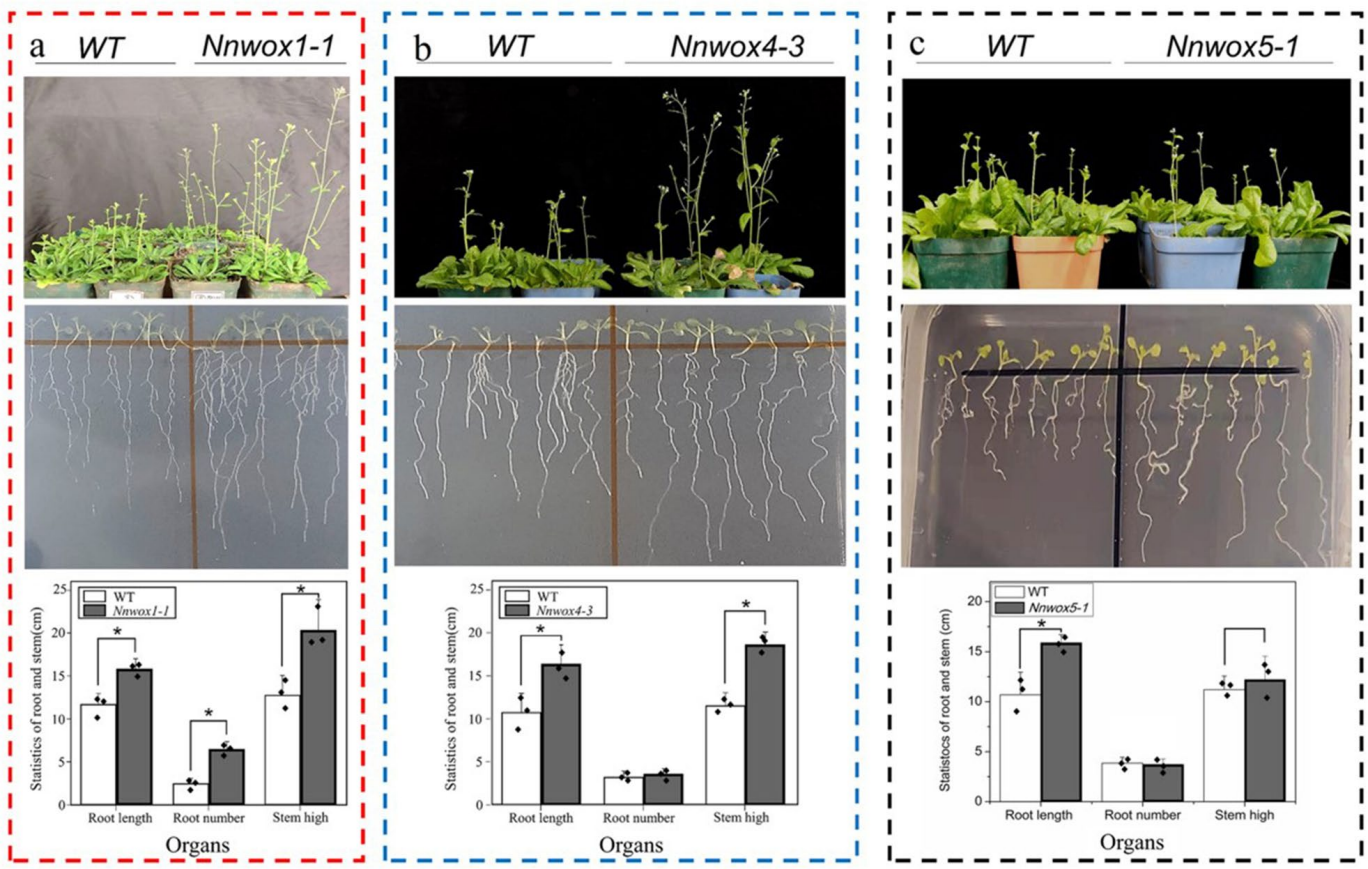
Functional analysis of *NnWOX1-1*, *NnWOX4-3*, and *NnWOX5-1* in transgenic *Arabidopsis* plants. **a** Assessment of stem and root development in transgenic plants with constitutive *NnWOX1-1* expression and wild-type plants. **b** Effect of *NnWOX4-3* on stem growth and root development in transgenic *Arabidopsis* plants. **c** Effect of *NnWOX5-1* on stem growth and root development in transgenic plants. The mean values were calculated from three replicate experiments, and error bars show standard deviation. Statistical analysis was performed using a Student's t-test, * *P* < 0.05

*pSN1301:NnWOX1-1*, *pSN1301:NnWOX4-3*, and *pSN1301:NnWOX5-1* were constructed and transferred to *Arabidopsis* plants to identify the gene functions. “Positive” plants were identified by PCR (Additional Fig. 2). Seeds of the T2 generation were sown on base material (V:V soil: vermiculite = 1:1) and Murashige and Skoog (MS) culture medium. Transgenic *NnWOX1-1* plants had longer stems than wild-type plants. Concurrently, the root quantity and length in transgenic *NnWOX1-1* plants were greater than those in wild-type plants (Fig. 4a). In addition, transgenic *NnWOX4-3* plants exhibited more roots, longer roots, and longer stems than wild-type plants (Fig. 4b). Although root length was greater in transgenic *NnWOX5-1* plants than in wild-type plants, root quantity and stem height showed no significant differences (*p* < *0.05*) between groups (Fig. 4c).

### Transcriptome analysis of transgenic and wild-type plants

**Fig. 5:**
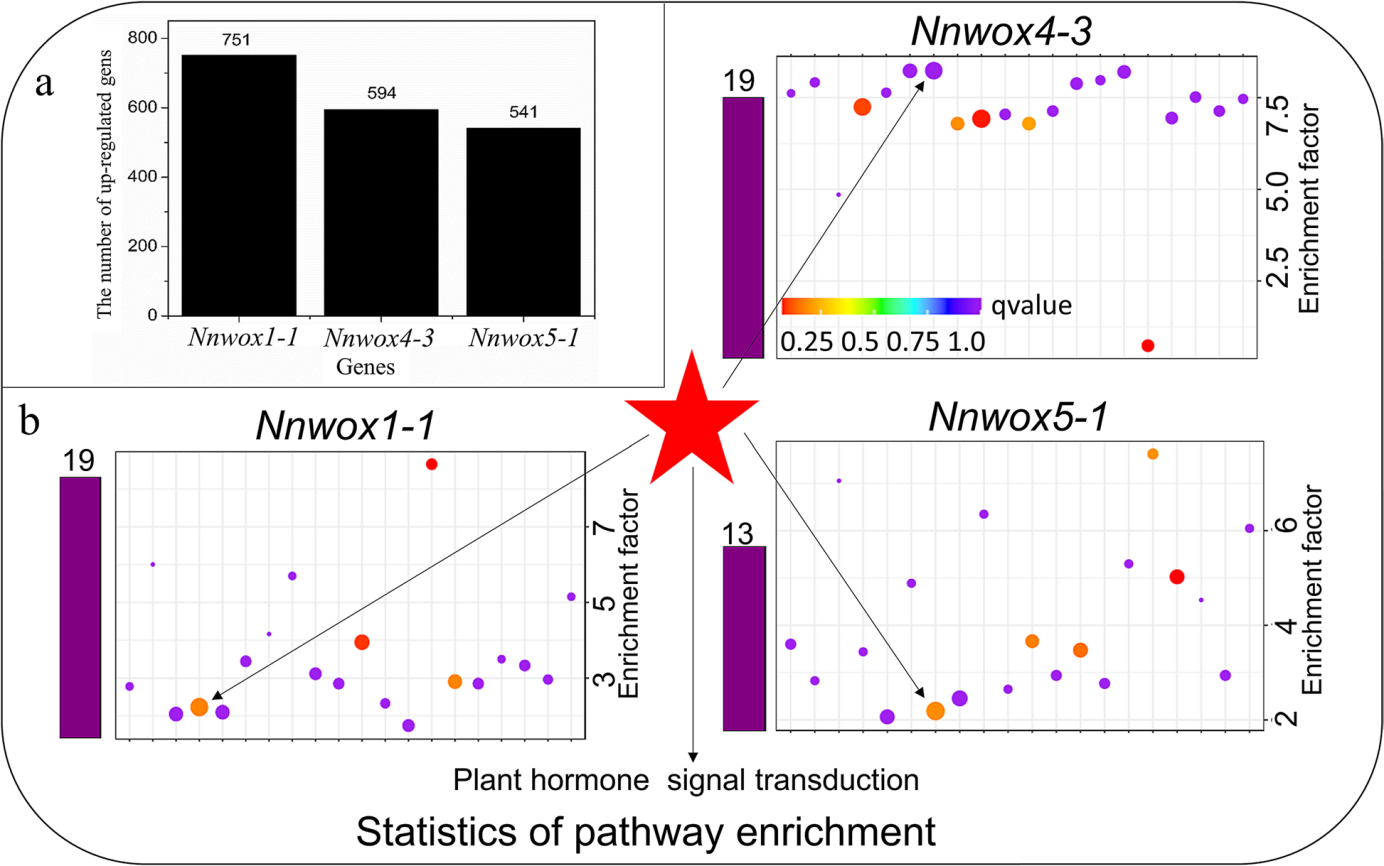
Statistical analysis of upregulated genes and pathway enrichment in transgenic plants with constitutive expression of *NnWOX1-1*, *NnWOX4-3*, and *NnWOX5-1*. **a** Number of upregulated genes following overexpression of *NnWOX1-1*, *NnWOX4-3*, and *NnWOX5-1* in *Arabidopsis* plants. **b** Genes involved in plant hormone transduction pathway are counted in transgenic plants expressing *NnWOX1-1*, *NnWOX4-3*, and *NnWOX5-1*

Transgenic *Arabidopsis* plants with* NnWOX1-1*, *NnWOX4-3*, and *NnWOX5-1* and wild-type plants at the six-leaf stage were analyzed using RNA sequencing (RNA-seq) to monitor changes in gene expression. A total of 751, 594, and 541 upregulated genes (Additional file 1) were found in transgenic *NnWOX1-1*, *NnWOX4-3*, and *NnWOX5-1* plants, respectively (Fig. 5a). These genes were classified into different biological pathways based on their functions. The most changes in gene expression occurred in general function prediction only, followed by signal transduction mechanism (Additional Fig. 3–1, 2, 3). The number of upregulated genes involved in plant hormone signal transduction pathways was the largest. There were 19, 19, and 13 upregulated genes involved in plant hormone signal transduction in the transgenic *NnWOX1-1*, *NnWOX4-3*, and *NnWOX5-1* plants, respectively (Fig. 5b). All genes related to the ethylene and IAA signal transduction pathways and their corresponding factors were selected (Table 1). Two genes involved in ethylene synthesis (*1-aminocyclopropane-1-carboxylate synthase* and *1-aminocyclopropane-1-carboxylate oxidase homolog 8*) were upregulated in all transgenic plants, which indicated that *NnWOX1-1*, *NnWOX4-3*, and *NnWOX5-1* affected ethylene synthesis. The expression of some ethylene and IAA response factors (*ethylene-responsive transcription factor ERF054*, *ethylene-responsive transcription factor ERF034*, *AP2/ERF and B3 domain-containing transcription repressor TEM1*, *ethylene-responsive transcription factor 14*, *AP2-like ethylene-responsive transcription factor SMZ*, *auxin-responsive protein IAA5*, *SAUR-like auxin-responsive protein*, *IAA-amino acid hydrolase ILR1-like 6*, *SAUR-like auxin-responsive protein*, and *IAA-amino acid hydrolase IAR3*) was also enhanced in the transgenic *NnWOX1-1*, *NnWOX4-3*, and *NnWOX5-1* plants (Table 1).

**Table 1:** Expression changes in genes involved in ethylene and IAA metabolism and response

ID	*Nnwox1-1*	*Nnwox4-3*	*Nnwox5-1*	Function annotation
**Ethylene metabolism and response factors**				
AT2G22810	3.53	3.63	3.86	1-aminocyclopropane-1-carboxylate synthase
AT5G67010	3.25	3.61	------	Ethylene-responsive transcription factor ERF121
AT4G28140	2.85	1.91	2.54	Ethylene-responsive transcription factor ERF054
AT4G32800	2.59	2.21	1.57	Ethylene-responsive transcription factor ERF043
AT2G20880	2.57	------	1.74	Ethylene-responsive transcription factor ERF053
AT2G44940	2.52	2.68	2.38	Ethylene-responsive transcription factor ERF034
AT5G13910	2.24	------	------	Ethylene-responsive transcription factor LEP
AT1G25560	2.10	2.06	2.09	AP2/ERF and B3 domain-containing transcription repressor TEM1
AT1G04370	2.01	2.08	2.03	Ethylene-responsive transcription factor 14
AT4G18450	1.83	------	------	Ethylene-responsive transcription factor ERF091
AT3G61400	1.53	2.25	1.44	1-aminocyclopropane-1-carboxylate oxidase homolog 8
AT3G50260	1.47	1.14	------	Ethylene-responsive transcription factor ERF011
AT2G23340	1.42	------	------	ERF/AP2 transcription factor DEAR3
AT3G54990	1.36	1.88	1.45	AP2-like ethylene-responsive transcription factor SMZ
AT5G57390	1.18	------	------	AP2-like ethylene-responsive transcription factor AIL5
AT1G22810	1.17	1.05	------	Ethylene-responsive transcription factor ERF019
AT5G67000	------	3.39	2.82	Ethylene-responsive transcription factor ERF122
AT1G68550	------	1.28	------	Ethylene-responsive transcription factor ERF118
AT5G07580	------	1.80	------	Ethylene-responsive transcription factor ERF106
AT4G27950	------	------	2.71	Ethylene-responsive transcription factor CRF4
AT5G25810	------	------	2.50	Ethylene-responsive transcription factor TINY
AT5G44210	------	------	2.07	Ethylene-responsive transcription factor 9
AT5G65100	------	------	2.05	Ethylene insensitive 3 family protein
**IAA metabolism and response factors**				
AT1G15580	4.52	3.82	3.33	Auxin-responsive protein IAA5
AT4G22620	2.64	1.95	1.45	SAUR-like auxin-responsive protein
AT1G44350	2.10	2.03	1.74	IAA-amino acid hydrolase ILR1-like 6
AT2G24850	2.49	------	------	Tyrosine aminotransferase 3
AT3G23030	1.77	1.81	------	Auxin-responsive protein IAA2
AT5G43890	1.66	------	------	Indole-3-pyruvate monooxygenase YUCCA5
AT1G51950	1.46	1.73	1.22	Auxin-responsive protein IAA18
AT1G75310	1.26	1.35	------	Auxin-like 1 protein
AT2G46690	1.26	1.96	1.50	SAUR-like auxin-responsive protein
AT1G51760	1.16	1.61	1.58	IAA-amino acid hydrolase IAR3
AT3G04730	------	------	1.61	Auxin-responsive protein IAA16
AT5G20820	------	3.65	3.60	SAUR-like auxin-responsive family protein

“------” represented no changes of expression

### Determination of ethylene, IAA, ABA, and lignin content

**Fig. 6:**
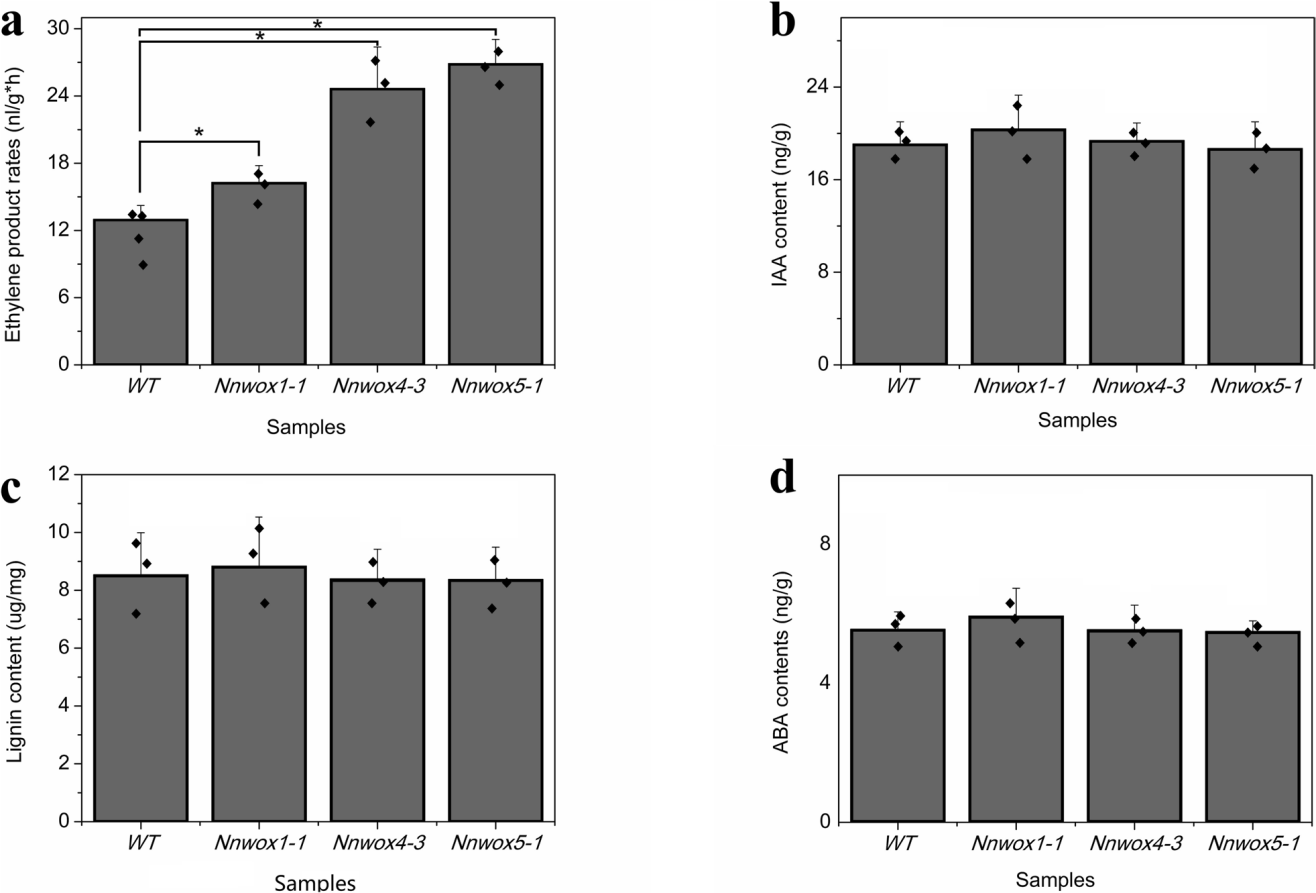
Determination of ethylene, IAA, lignin, and ABA contents in transgenic plants expressing *NnWOX1-1*, *NnWOX4-3*, and *NnWOX5-1* and in wild-type plants. **a** Ethylene content in six-leaf-stage transgenic and wild-type plants. **b** Analysis of IAA content in transgenic and wild type plants at the six-leaf stage. **c** Lignin contents of the three transgenic and wild-type plants were analyzed to monitor the effect of *NnWOX1-1*, *NnWOX4-3*, and *NnWOX5-1* expression. **d** ABA content in *NnWOX1-1*, *NnWOX4-3*, and *NnWOX5-1* transgenic and wild-type plants. For statistical analysis, the data were recorded as means ± SEs of three experiments with approximately 10 seedlings in each experiment. “*” indicates that values are significantly different between samples (*P* < 0.05)

Ethylene, IAA, ABA, and lignin strongly influence AR formation in lotus plants; therefore, the levels of these compounds were measured in this experiment. Ethylene production rates were higher in *NnWOX1-1*, *NnWOX4-3*, and *NnWOX5-1* transgenic plants than in wild-type plants (Fig. 6a), indicating that *NnWOX1-1*, *NnWOX4-3*, and *NnWOX5-1* affected ethylene synthesis in transgenic plants. We also found that the IAA, ABA, and lignin contents in transgenic plants showed no significant differences compared to those in wild-type plants. The promotion of root development, stem growth, and flowering in transgenic plants expressing *NnWOX1-1*, *NnWOX4-3*, and *NnWOX5-1* was probably related to ethylene synthesis (Fig. 6b–d).

### Role of ethylene in AR formation and seedling growth in lotus plants

**Fig. 7:**
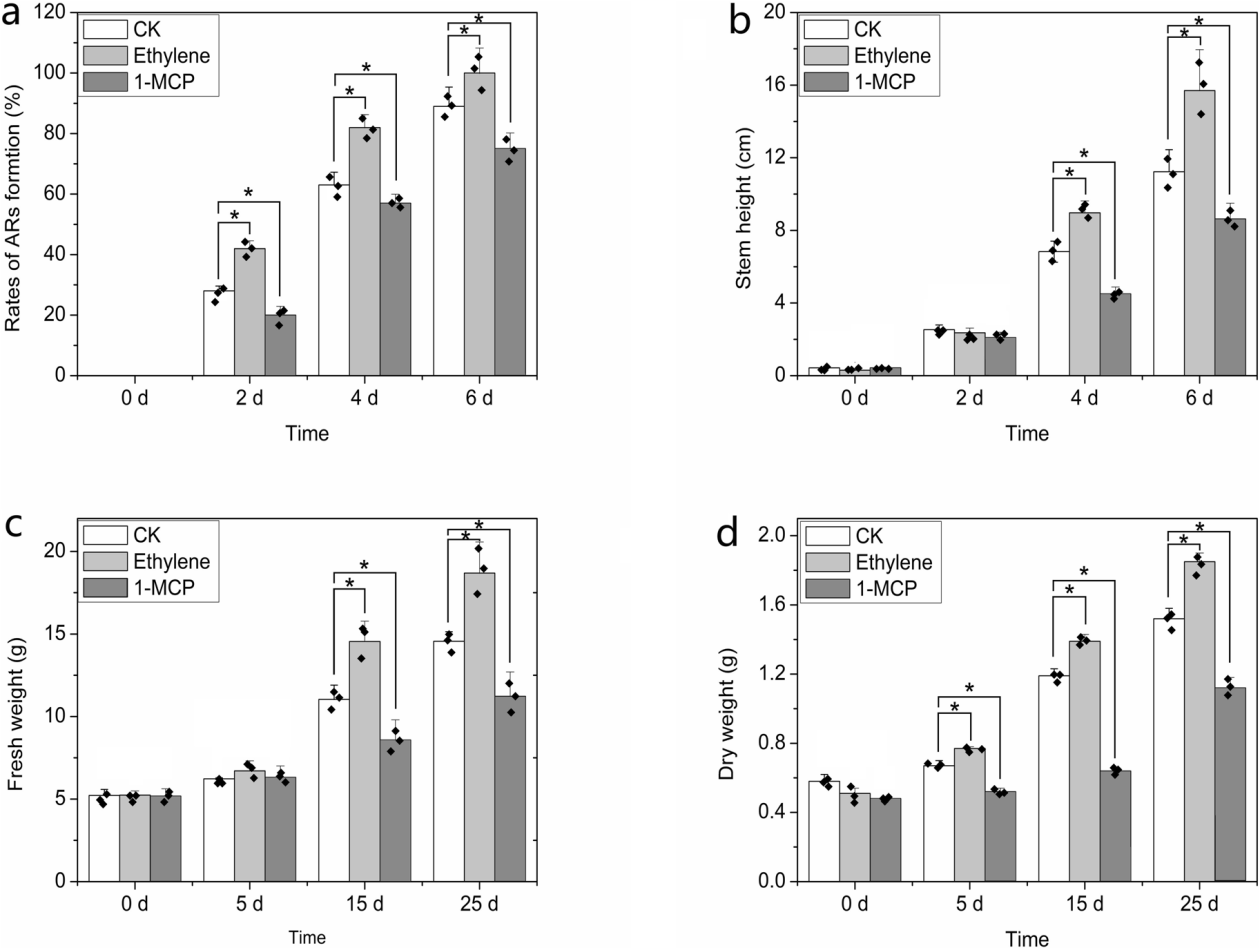
Role of ethylene in the development of lotus seedlings. **a** Effect of ethylene on the rates of adventitious root formation in lotus seedlings at 0, 2, 4, and 6 d after ethephon treatment. **b** Stem growth at 0, 2, 4, and 6 d after ethephon treatment in lotus seedlings. **c** Determination of fresh weight in response to ethephon treatment at 0, 5, 15, and 25 d in lotus seedlings. **d** Change in dry weight of lotus seedlings after ethephon treatment for 0, 5, 15, and 25 d. Each experiment was carried out with three replicates, and the data represent means ± SEs for approximately 20 seedlings. “*” indicates values that are significantly different between samples (*P* < 0.05)

Lotus seedlings were treated with 300 mg/L of ethephon and 1-methylcyclopropene (1-MCP). The rate of AR emergence was significantly increased in the seedlings treated with ethephon and was dramatically inhibited following the application of 1-MCP. The rate of AR emergence under ethephon treatment reached an approximately maximum value (100%) at 6 d, whereas a rate of only 65% was observed in the 1-MCP treatment group (Fig. 7a). In addition, the stems of seedlings treated with ethephon were longer than those of the control and 1-MCP treatment group plants (Fig. 7b). Further experiments indicated that ethephon probably affected the accumulation of metabolites in lotus seedlings. Ethephon significantly promoted fresh and dry weights, and the highest fresh and dry weights were found in plants treated with ethephon for 25 d (Fig. 7c, d), which indicated greater availability of metabolites and energy during AR formation.

### Adaptation of transgenic *Arabidopsis* plants to drought and salt stress

**Fig. 8:**
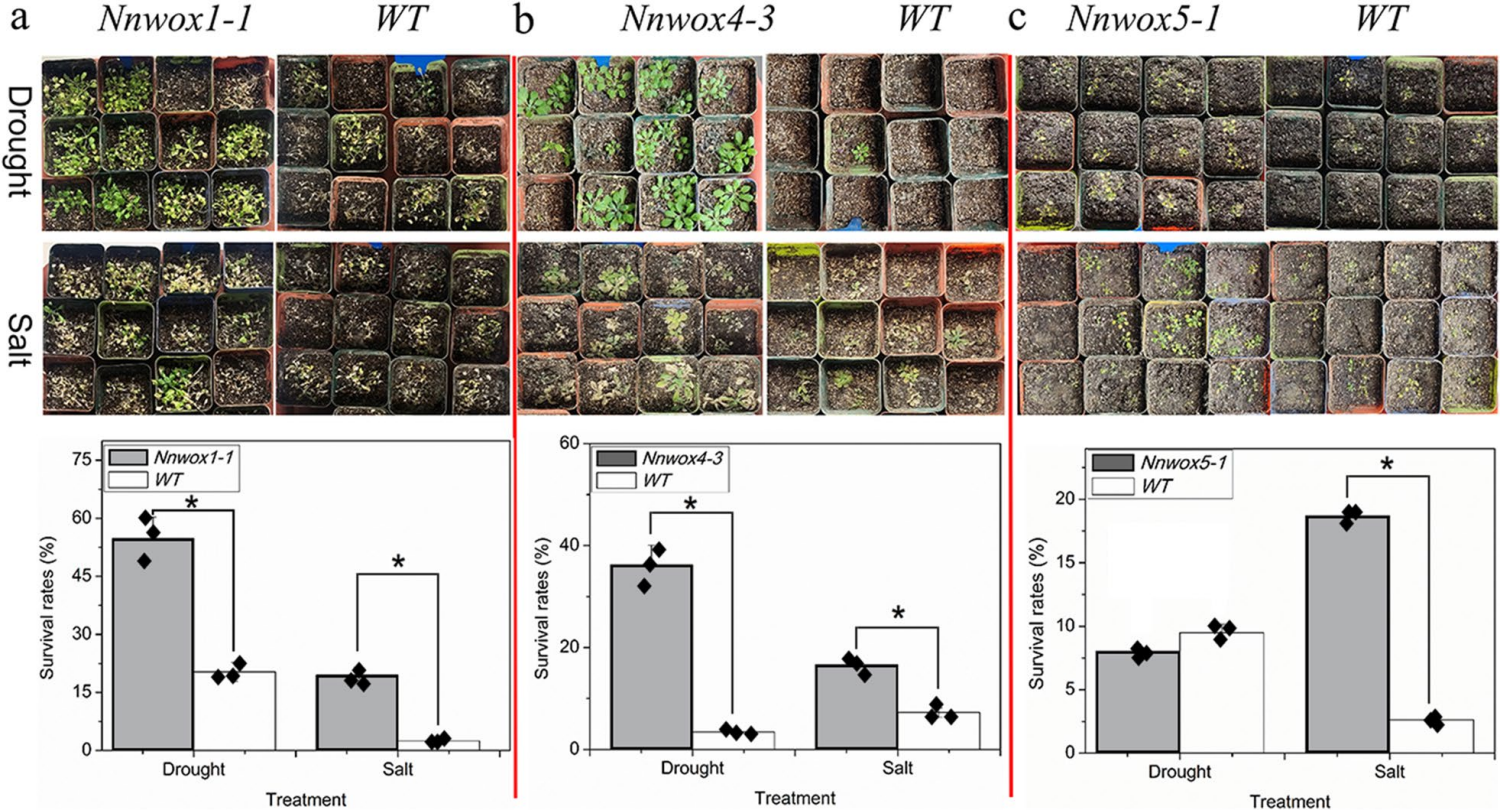
Survival rates of transgenic *NnWOX1-1*, *NnWOX4-3*, and *NnWOX5-1* plants and wild-type plants in response to drought and salt stress. **a** Survival rates of transgenic *NnWOX1-1 Arabidopsis* plants. **b** Survival rates of transgenic *NnWOX4-3* plants and wild-type plants. **c** Survival rates of *NnWOX5-1* transgenic plants. Data are presented as the means ± SE of three replicates, and *p* < *0.05* was accepted as the level of significance in statistical comparisons

Plants constitutively expressing *NnWOX1-1*, *NnWOX4-3*, and *NnWOX5-1* were subjected to drought and salt stress to evaluate the functions of these three genes under adverse environmental conditions. Overexpression of *NnWOX1-1*, *NnWOX4-3*, and *NnWOX5-1* improved the responses of transgenic plants to salt stress when compared with those of wild-type plants (Fig. 8a–c). In addition, *NnWOX1-1* and *NnWOX4-3* improved adaptation to drought resistance in the transgenic plants (Fig. 8a, b). Further analysis was performed to monitor gene expression in the three transgenic plant varieties. The expression of some stress-related genes (such as the *MYB*, *WRKY*, and *N-acetylcysteine* (*NAC*) transcription factors; and *dehydration responsive element binding protein* (*DREB*) and *late embryogenesis abundant* (*LEA*) proteins) was found to change in the transgenic plants when compared with that in the wild-type plants (Table 2). Therefore, the enhanced drought and salt stress tolerance observed in transgenic plants might be derived from the change in the expression of these genes.

**Table 2:** Expression of genes related to stress responses in transgenic *NnWOX1-1*, *NnWOX4-3*, and *NnWOX5-1 Arabidopsis* plants

ID annotation	*Nnwox1-1*	*Nnwox4-3*	*Nnwox5-1*	Function
AT5G11590	4.11	4.56	4.12	Dehydration-responsive element-binding protein 3
AT2G32460	3.41	3.16	------	MYB domain protein
MSTRG.4188	3.15	2.84	2.88	WRKY DNA-binding protein
AT1G66550	3.16	2.83	2.88	WRKY transcription factor 67
AT3G27810	2.99	3.60	2.82	Transcription factor MYB21
AT4G34990	2.28	2.08	1.28	Transcription factor MYB32
AT4G04710	2.23	4.15	4.08	Calcium-dependent protein kinase 22
AT2G36590	1.85	1.74	2.24	Proline transporter 3
AT1G80590	1.83	1.92	1.91	WRKY transcription factor 66
AT5G52660	1.72	1.60	------	MYB family transcription factor
AT5G38710	1.66	1.57	------	Proline dehydrogenase 2
AT4G05100	1.54	1.68	1.67	MYB domain protein 74
AT3G18400	1.37	1.42	1.30	NAC domain containing protein 58
AT1G14600	1.37	1.38	------	Putative myb family transcription factor
AT4G31000	1.12	1.03	------	Calmodulin-binding protein
AT4G05020	1.64	1.59	1.67	Calcium-transporting ATPase 8
AT3G21070	2.17	1.82	------	NAD(H) kinase 1
AT2G41280	5.28	------	------	Late embryogenesis abundant protein M10
AT4G08400	------	------	4.90	Proline-rich extensin-like family protein
AT1G54970	------	------	3.03	Proline-rich protein 1
AT5G5437	2.89	------	------	Late embryogenesis abundant protein-like protein
AT5G61620	------	4.03	3.71	MYB-Like transcription factor family protein
AT5G26660	1.8	2.0	------	Transcription Factor MYB86
AT2G40750	------	------	1.59	WRKY DNA-binding protein 54
AT5G56840	------	2.13	1.50	MYB-like transcription factor family protein

“------” represented no changes of expression

## Discussion

ARs are responsible for water and nutrient uptake and are necessary for plant growth, especially when principal roots are not thoroughly developed; thus, the developmental process of ARs affects the yield, quality, and adaptation of plants to the environment. Members of the *WOX* family are essential transcription factors that regulate various biological processes in plants. *WOX*s influence the apical meristem of roots, and the overexpression of these genes results in changes in root development [45]. For example, transgenic *WOX11* and *WOX12* plants exhibit more roots than non-transgenic plants [46, 47]. In a previous study, we screened transcripts from the entire genome of the lotus (*Nelumbo nucifera* Gaertn) and observed that the expression of some *NnWOXs* was induced by IAA and sucrose [23, 24]. In the present study, *NnWOX1-1*, *NnWOX4-3*, and *NnWOX5-1* were cloned using RT-PCR (Fig. 1).

The *WOX* family is distributed among monocots and dicots, and several *WOX*s with superfamily homeodomains have been analyzed in detail within the past year. *WOX*s are grouped into three clades: ancient (distributed in algae and higher plants), intermediate (distributed in vascular plants), and modern (distributed in seed plants) [48, 49]. Among the 14 *WOX* proteins of *Arabidopsis*, *WOX1-7* are classified into the modern clade; *WOX8*, *9*, *11*, and *12* belong to the intermediate clade; and *WOX10*, *13*, and *14* belong to the ancient clade. The same distribution was also observed in an analysis of the gene structure of *WOX*s in poplars. According to the reports above, *NnWOX1-1*, *NnWOX4-3*, and *NnWOX5-1* belong to the modern clade. Most *WOXs* in the modern clade have a homeodomain, a feature of the gene structure of this family in *Arabidopsis* [45]. In this study, we found that *NnWOX1-1* and *NnWOX4-3* contained a homeobox, whereas *NnWOX5-1* contained a homeodomain. Therefore, *NnWOX1-1* and *NnWOX4-3* showed higher similarity to each other than to *NnWOX5-1*, which was further confirmed by the phylogenetic analysis of these three genes (Fig. 1). The homeodomain of Cucurbitaceae *WOXs* is highly conserved, suggesting that these genes have a close evolutionary history [50]. However, the lack of a homeodomain in *NnWOX1-1* and *NnWOX4-3* indicates that different functions might have existed in *NnWOX1-1* and *NnWOX4-3* than in *NnWOX5-1*. Members of the *WOX* family are diverse in gene structure, and the variation usually results in significant changes in biological processes. In our study, we found that 10 motifs were present in the three genes, with eight, five, and seven motifs included in *NnWOX1-1*, *NnWOX4-3*, and *NnWOX5-1*, respectively. This indicated that the functions of these three genes might be different in metabolic regulation mechanisms in lotus plants (Fig. 2).

Ethylene, a critical plant hormone, controls several metabolic processes, from seed germination to flowering. Ethylene positively regulates AR formation by controlling epidermal cell death before AR emergence [14]. In petunia cuttings, the development of ARs is regulated by ethylene [51]. The IAA signal is also strictly required for AR formation, as has been confirmed in lotus plants [23]. Sucrose also influences root development [52]. A further analysis of the effect of sucrose on AR formation showed that this effect mainly occurs during the induction period of AR development [53]. In lotus plants, the role of sucrose in AR formation has been assessed, and sucrose, like IAA, participates in AR development [25]. In the present study, the three genes were induced by IAA and sucrose, although their expression profiles were different. In addition, the upregulation of mRNA levels of *NnWOX1-1*, *NnWOX4-3*, and *NnWOX5-1* varied in different organs (Fig. 3), suggesting that their functions in biological pathways might be different. This phenomenon is also found in *Salix suchowensis*; a total of 15 *WOX*s show organ-specific expression profiling, although some genes are classified into the same subgroup [54]. The transcriptional levels of *WOXs* in strawberry vary in roots, stems, and leaves, suggesting that these genes likely influence many developmental processes during plant growth by affecting gene expression [55].

The functions of *NnWOX1-1*, *NnWOX4-3*, and *NnWOX5-1* were explored in *Arabidopsis* plants. We observed that transgenic plants expressing *NnWOX1-1* and *NnWOX4-3* had longer stems, more roots, and longer roots than wild-type plants, whereas the overexpression of *NnWOX5-1* only improved root length (Fig. 4). *WOX1* influences lateral organ formation [56], which has been further confirmed in *Medicago truncatula* [57]. A recent report stated that *WOX1* can interact with auxins to regulate the development of leaf veins in cucumber plants [58]. Together with our findings, this suggests that *WOX1* has multiple roles in plant growth. *WOX4* is related to cytokinins and is required for meristem maintenance in rice [59]. In *Arabidopsis*, *WOX4* influences the diverse functions of stem cells and procambium development [60]. *PtWOX4* influences the auxin signal transduction pathway and regulates cell division in the stem [61]. In the present study, transgenic plants expressing *NnWOX4-3* had enhanced root development and longer stems than wild-type plants (Fig. 4). We also found that the expression of many genes related to signal transduction mechanisms was changed in the three transgenic plants (Additional Fig. 3 - 1, 2, 3). Further analysis showed that genes involved in ethylene synthesis and response were upregulated (Table 1, Fig. 5). In addition, it was shown that the rates of ethylene synthesis were higher in the transgenic plants than in the wild-type plants (Fig. 6). This suggested that *NnWOX1-1*, *NnWOX4-3*, and *NnWOX5-1*, which promoted root development, were probably related to ethylene biosynthesis. Exogenous ethylene improves endogenous ethylene accumulation and auxin transport [21, 62], and auxin can also mediate ethylene to control AR formation in sunflowers [63]. Some important physiological indices showed that the lotus seedlings treated with ethephon in this study had increased AR emergence rates, stem heights, and fresh and dry weights (Fig. 7). Therefore, we concluded that ethylene signaling critically influences AR formation in lotus plants.

Responding to adverse environmental factors is very common for most members of the *WOX* family, suggesting that the expression of *WOX*s can improve plant adaptation to stress. Overexpression of *OsWOX13* could improve drought tolerance in rice [64]. Several *WOXs* in cotton have been identified, and virus-induced gene silencing showed that these genes play a critical role in plant growth by responding to drought stress [65]. Han et al. reported that *CsWOX*, which is involved in the phytohormone pathway, plays multiple roles in plant growth and is responsive to stress [66]. The same phenomenon was found in our study, and constitutive expression of *NnWOX1-1*, *NnWOX4-3*, and *NnWOX5-1* resulted in an improvement in salt tolerance in transgenic *Arabidopsis* plants (Fig. 8). However, further analysis was required to determine whether ethylene signaling affected drought and salt adaptation. Some stress-related genes, including *DREB*, *MYB*, *WRKY*, *NAC domain containing protein*, and *LEA* were found to have enhanced expression in the transgenic plants (Table 2). In previous studies, these genes have been shown to improve stress tolerance [67–70]. We believe that *NnWOX1-1*, *NnWOX4-3*, and *NnWOX5-1* improve drought and salt tolerance in *Arabidopsis* plants probably by regulating expression of the abovementioned genes. Lignin synthesis strongly influences AR development via the sucrose pathway [26]. In this study, we found that the overexpression of *NnWOX1-1*, *NnWOX4-3*, and *NnWOX5-1* did not affect lignin content in *Arabidopsis* plants, suggesting that the change in root growth did not result from the synthesis of IAA, ABA, and lignin. In summary, our findings provide primary insights into the roles of *NnWOX1-1*, *NnWOX4-3*, and *NnWOX5-1*. Further exploration of the regulatory network of AR formation and stress responses to lotus WOXs is necessary.

## Conclusion

*NnWOX1-1*, *NnWOX4-3*, and *NnWOX5-1* were cloned based on transcriptional information regarding AR development in lotus seedlings. The structures, phylogenetic relationships, motifs, and chromosomal locations were analyzed in detail. These three genes were induced by the exogenous application of IAA and sucrose. The constitutive expression of *NnWOX1-1*, *NnWOX4-3*, and *NnWOX5-1* significantly promoted root development and stem growth in transgenic plants. Plant responses to stresses were also identified following transgene transfection in *Arabidopsis*. Further experiments confirmed that ethylene production rates in the transgenic plants were dramatically higher than those in wild-type plants. IAA, ABA, and lignin levels did not change, suggesting that *NnWOX1-1*, *NnWOX4-3*, and *NnWOX5-1* regulated root formation in transgenic *Arabidopsis* plants probably through the biosynthesis of endogenous ethylene. We also found that ethylene had a positive effect on AR formation and growth in lotus seedlings. Our findings are helpful in further exploring *WOX* functions and providing a better understanding of AR formation in lotus plants.

## Materials and methods

### Preparation of plant materials

Lotus seeds (Taikong Lotus 36) were used to conduct experiments in this study. Seeds were obtained from The Guangchang Bailian Institute and cultivated in an experimental field of aquatic vegetables at Yangzhou University, Southeast China. For plant growth, the field was maintained at a 20–25 cm water depth in the spring, with a 40–50 cm water depth in the summer. The temperature was 25–35 °C during the day and 18–25 °C at night throughout the entire growing season (from April to October). Seeds were harvested in November and stored at room temperature.

### Cloning of *NnWOX1-1*, *NnWOX4-3*, and *NnWOX5-1*

The full sequences of the three genes were obtained from the NCBI database. The following primers were used: *NnWOX1-1*, forward primer: 5′-ATGGAAAGGAAAGATGAT-3′, reverse primer: 5′-TCACTTGGATTTGAATGG-3′. *NnWOX4-3*, forward primer: 5′-ATGGAGGGCAAAGAGGAG-3′, reverse primer: 5′-TTACACTCTGGCCTTGAA-3′. *NnWOX5-1*, forward primer: 5′-ATGGGGAAGGACGTTGAG-3′, reverse primer 5′-TTAGACGTGAGGGTTGCT-3′. Total RNA was extracted using an RNeasy MinElute Cleanup Kit (QIAGEN, Germany), following the supplied instructions. The first cDNA strand was synthesized using 1–2 µg of total RNA following the elimination of DNA contamination. A 20 µL mixture containing 2.5 µL dNTP, 2 µL each of forward and reverse primers, 2.5 µL MgCl_2_, 2 µL Taq polymerase (5 U), 2 µL cDNA fragments, and 7 µL dH_2_O was utilized to perform PCR component analysis. PCR was performed using the following program: 1 min at 94 °C for initial denaturation; 35 cycles of 1 min at 94 °C for denaturation, 1 min at 56–60 °C for annealing, and 1 min at 72 °C for extension; and 10 min at 72 °C for the final extension. A GeneJET Gel Extraction Kit (Thermo, Germany) was used to extract target gene fragments, which were ligated into the cloning vector (pMD 18-T vector; TaKaRa). These ligated vectors were grown in DH5α *Escherichia coli* and sequenced by Sangon Biotechnology Co., Ltd. (Shanghai, China).

### Sequence analysis

A comparison among these three genes was performed using the DNAman and Simple Modular Architecture Research Tool (SMART) software programs to analyze the conserved domains. *NnWOX1-1-*, *NnWOX4-3-*, and *NnWOX5-1*-encoded proteins were selected, standardized to fasta format, and then successively imported into the software channels. In the option section, each sequence marked by black color was set to represent homology. A phylogenetic tree was constructed using DNAman and MEGA X software. In addition, the conserved motifs of the *NnWOX1-1-*, *NnWOX4-3-*, and *NnWOX5-1*-encoded proteins were analyzed using the online DNAman and MEME server v5.4.1 software programs (http://meme-suite.org/tools/meme). First, standardized formats of *NnWOX1-1-*, *NnWOX4-3-*, and *NnWOX5-1*-encoded proteins were generated using DNAman software and then input into the data submission system of MEME software according to the instructions. Second, “zero-or-one occurrence per sequence (zoops)” and “ten” motifs in the required box were set in the option system. Finally, the output was visualized by Btools software. Chromosomal location maps of the three genes were constructed using DNAman and TBtools software. The detailed process has been described by Chen et al. [71].

### Gene expression analysis

The RT-qPCR method was used to monitor the expression of the three genes in lotus seedlings treated with 20 g/L sucrose and 10 µL IAA. In addition, the ARs, leaves, stems of six-leaf seedlings, and flowers were selected to analyze organ-specific expression characteristics. Total plant RNA was extracted using an RNeasy MinElute Cleanup Kit (QIAGEN) according to the manufacturer's instructions. Genomic DNA was removed using DNase I, and then 3 µg RNA was used to synthesize first-strand cDNA with a RevertAid First Strand cDNA Synthesis Kit (Fermentas, USA). SuperReal PreMix Plus (Tiangen, China) was used to calculate the mRNA levels of *NnWOX1-1*, *NnWOX4-3*, and *NnWOX5-1* with three biological replicates on an Mx 3000P machine (STRATAGENE, http://www.stratagene.com). The forward primer for *NnWOX1-1* was 5′-GGTGGTGATTGTGAAGGAGT-3′, and the reverse primer was 5′-CAATCCAACGCCCTTACTAT-3′. The forward primer for *NnWOX4-3* was 5′-TACTGTGGGTATTCAGGGCA-3′, and the reverse primer was 5′-TGACTCTTCTGGAAACCCTT-3′. The forward primer for *NnWOX5-1* was 5′-CGGCTGTAACCTTTGGACTT-3′, and the reverse primer was 5′-TCCCAGGGCAGTTCCTTTTG-3′. Lotus *β-actin* was used as an internal standard, with the forward primer 5′-AACCTCCTCCTCATCGTACT-3′ and the reverse primer 5′-GACAGCATCAG CCATGTTCA-3′. The mRNA level was calculated using the 2^−△△Ct^ method [21]. A 25 μL reaction mixture containing 12.5 μL SYBR Premix Ex Taq II (Tli RNaseH Plus) (2 ×); 1 μL of each primer (forward and reverse); 3 μL cDNA of *NnWOX1-1*, *NnWOX4-3*, and *NnWOX5-1*; and 8.5 μL dH_2_O was prepared. The PCR program comprised 30 s at 94 °C, followed by 40 cycles of 5 s at 95 °C and then 60 s at 60 °C.

### Gene function analysis of *NnWOX1-1*, *NnWOX4-3*, and *NnWOX5-1*

*NnWOX1-1*, *NnWOX4-3*, and *NnWOX5-1* were ligated into the pGEM-T vector (cloning vector) and sequenced by Sangon Biotechnology Co., Ltd., after being transferred into *E. coli* to amplify the plasmids. The three genes were removed from the cloning vector by digestion with *Bam*HI and *Kpn*I restriction enzymes and transferred into a plant transformation vector (pSN1301) containing the CaMV 35S promoter. After completing the vector construction process, pSN1301:: *NnWOX1-1*, pSN1301:: *NnWOX4-3*, and pSN1301:: *NnWOX5-1* were transferred into *Agrobacterium tumefaciens* strain GV3101 for infection preparation. Wild-type *Arabidopsis* plants (Columbia) were transformed using the floral dip method [72]. The seeds of the T0 generation were screened using 20 μg/g hygromycin B on MS medium after sterilization. The screened plants were cultivated in a greenhouse at 22 °C under a 12 h photoperiod. Positive plants were further identified using RT-PCR. The primers, mixtures, and PCR processes for these three genes were the same as those used for gene cloning.

The T2 generation seeds of transgenic and wild-type plants at the six-leaf stage were selected for functional identification. First, the transgenic and wild-type seeds were sterilized with 70% alcohol for 10 s, and then sodium hypochlorite (10%) was added for 25 min. The seeds were placed on the medium and base material (soil:vermiculite, 1:1, v/v) for further analysis of plant phenotypes and root development.

### RNA-seq analysis

#### RNA-seq of *NnWOX1-1*, *NnWOX4-3*, and *NnWOX5-1*

Transgenic and wild-type seeds were placed on the base material for germination and then transferred to a light house. The temperature was maintained at 22–23 °C with a 12 h photoperiod. The roots of six-leaf-stage plants were used for gene expression analysis by RNA-seq. Approximately 2–3 μg RNA of transgenic and wild-type plants was prepared for library construction. The detailed process for library construction is described by Cheng et al. [24], and sequencing was performed by Nanjing Jisi Huiyuan Biotechnology Co., Ltd., using a special construct.

### Identification and annotation of differentially expressed genes (DEGs)

The samples were evaluated in parallel using Illumina gene expression sample preparation kits. DEGs were screened using the NOISeq method, and the detailed protocol was previously described by Cheng et al. [23]. The thresholds of the DEGs were fold change ≥ 2 and divergence probability ≥ 0.8. The genes obtained in this experiment were annotated using the Gene Ontology (GO) tool with three ontologies such as molecular function, cellular component, and biological process. All the DEGs were enriched and classified into various biological functions after comparison with the lotus genome obtained from the NCBI database. All the DEGs were compared to the GO database (http://www.geneontology.org/), and the number of genes was calculated for the three ontologies mentioned above. Further, these DEGs were then input into a list of significantly enriched GO terms determined by a hypergeometric test. For pathway (biological functions) analysis, the Kyoto Encyclopedia of Genes and Genomes (KEGG) tool was used for the organized enrichment analysis of DEGs. Specifically, all identified DEGs were grouped into different biological pathways following comparison with the genome data derived from the NCBI database using KEGG. Therefore, all the DEGs were grouped into different metabolic pathways.

### Ethylene, IAA, and ABA identification

The transgenic *NnWOX1-1*, *NnWOX4-3*, *NnWOX5-1*, and wild-type seeds were cultivated on the base material and transferred into an illuminated incubator at 22 °C. Six-leaf plants were selected for ethylene, IAA, and ABA identification. First, three six-leaf-aged seedlings from each sample were washed and placed in a bottle tightly sealed with a plastic plug. These bottles were transferred to a dark carton for 10 d at 25 °C. The ethylene production rate was measured using a gas chromatograph (Agilent, USA) according to the procedure outlined by Fiserova et al. [73]. This experiment was repeated thrice. Then, 20 seedlings of the transgenic and wild-type plants were frozen in liquid nitrogen and ground into powder with a rod. Approximately 2 g of powdered sample from each plant was transferred into approximately 600 µL of isopropyl alcohol/water/concentrated hydrochloric acid (2:1:0.002 v/v/v) for IAA and ABA extraction. These mixtures were placed into an agitator for 25–30 min at 4 °C; 1 mL dichloromethane was added, and the shaking was repeated for 30 min at 4 °C. The mixtures were centrifuged at 10,000 rpm (5180 × g) for 5 min at 4 °C. The lower liquid layer was collected and dried under nitrogen. The dry powder was dissolved in 100 mL filtered methanol, and 50 mL of the resulting solution was subjected to liquid chromatography (Sigma, Germany) for IAA content determination [24]. ABA content was determined using the method reported by Zdunek and Lips [74].

### Lignin identification

Thirty transgenic *NnWOX1-1*, *NnWOX4-3*, and *NnWOX5-1* and wild-type plant roots were selected for polymer content analysis. The plant growth conditions were the same as those described in the “Gene function analysis of *NnWOX1-1*, *NnWOX4-3*, and *NnWOX5-1*” section. The plant roots were dried at 60 °C for approximately 2 d, and 60 mg dry power was transferred into ethanol (1 mL, 70%). The mixture was vortexed and centrifuged at 10,000 rpm at room temperature. The supernatant was discarded, and the precipitate was washed with 1 mL of acetone. A 1 mL solution of chloroform/methanol (1:1 v/v) was added to the precipitate after the supernatant was discarded. The precipitate was dried under vacuum. The precipitate was treated with 1.5 mL of 0.1 M sodium acetate buffer (pH 5.0) at 80 °C for approximately 30 min and then transferred into a tube with 10 µL of 0.01% sodium azide, amylase, and pullulanase and incubated at 37 °C overnight. After heating at 100 °C for a few minutes, the precipitate was washed with water multiple times. One milliliter of acetone was added to the sample, which was then dried via vacuum. The precipitate was dissolved in 100 µL of 25% acetyl bromide for 3 h at 50 °C. Four hundred microliters of 2 M sodium hydroxide and 70 µL of 0.5 M hydroxylamine hydrochloride were added. Acetic acid was added to increase the volume to 2 mL, and 200 µL of the supernatant was analyzed in a multifunctional microplate reader (1510–04201, Semefi, USA) to determine its absorbance at 280 nm. The polymer lignin content was calculated using the following formula:

$$\mathrm{Lignin}\,\mathrm{content}=\frac{\mathrm{ABS}}{\mathrm{Coeff}\times0.539\mathrm{cm}}\times\frac{2\mathrm{ml}}{\mathrm{Weight}}\times100\%$$

(ABS, absorbance value; Coeff , absorption coefficient)

### Stress responses for transgenic and wild-type plants

Approximately 50 seeds of transgenic and wild-type plants were placed in water for 24 h and then sown on the medium. The detailed cultivation conditions were the same as those described in “Materials and Methods section: Gene function analysis of *NnWOX1-1*, *NnWOX4-3*, and *NnWOX5-1*”. Six-leaf seedlings of wild-type and transgenic plants were subjected to drought and salt stress. For the drought treatment, the plants were first treated with a unified water management scheme, and then water was withdrawn for approximately 10 d. Survival rates were determined after 7 d of water recovery. For the salt treatment, 100 mM NaCl was used to treat transgenic and wild-type plants, and survival rates were determined after 10 d. For the survival rate, the maintenance of plant growth (some leaves remained green) or the occurrence of new leaves was used as a criterion for survival, and withering of plants during the recovery period was considered death. Three biological replicates were performed for each experiment, and at least 50 seedlings were used for each replicate.

#### Role of ethephon in AR formation and lotus plant growth

Approximately 300 lotus seeds were selected to identify AR formation rates. The seed coat was broken before treatment. Approximately 50 seedlings were first treated with 300 mg/L ethephon and 300 mg/L 1-MCP (1-MCP can bind ethylene receptors, preventing ethylene from affecting plant growth) for 2 d, and then transferred to water for continuous cultivation. For AR abundance analysis, 20 lotus seedlings cultivated for 0, 2, 4, and 6 d were selected to monitor AR formation. ARs with a length of≧0.2 cm after breaking through the hypocotyl were used to determine abundance. For stem length analysis, lotus seedlings cultivated for 0, 2, 4, and 6 d were selected to monitor growth status. A ruler graduated in centimeters was used to measure the stem length (from the hypocotyl to the bottom edge of the first leaf). For fresh and dry weight assessments, 20 seedlings cultivated for 0, 5, 15, and 25 d in water were used to monitor the change in growth. The surface water on the seedlings at each time point was first removed with absorbent material before weighing, and then the seedlings were dried in an oven at 60 °C. The dry weight was recorded after no change in the weight of the dried material was observed. Three replicates were carried out in the above experiments, including analysis of AR abundance, stem length, and fresh and dry weight determinations.

#### Statistical analysis

Statistical analyses were performed using Origin Pro 8.0 (Origin Inc., Massachusetts, USA). For the analysis in each experiment, three repeated experiments were carried out and the means ± SE of three repetitions were determined. The means with standard deviations are displayed in all figures. Significant differences were determined by Student's t-test and differences at *p* < *0.05* were accepted as the level of significance (*, *p* < 0.05).

### Supplementary Information

**Additional file 1: Table S1.**

**Additional file 2: Fig. 1.**

**Additional file 3: Fig. 2.**

**Additional file 4: File 1.**

**Additional file 5: Fig. 3.**

## Data Availability

The material for all experiments was supported by the Aquatic Vegetable Lab of Yangzhou University. The collection of seeds complied with local and national guidelines and permissions were obtained. Detailed data have been deposited in the NCBI database (Project: PRJNA977099; CK1, CK2, CK3: SRR24775841, SRR24775840, SRR24775837; wox1-1, wox1-2, wox1-3: SRR24775836, SRR24775835, SRR24775834; wox4-1, wox4-2, wox4-3: SRR24775833, SRR24775832, SRR24775831; wox5-1, wox5-2, wox5-3: SRR24775830, SRR24775839, SRR24775838).
