# Supplementary material for: NnWOX1-1, NnWOX4-3, and NnWOX5-1 of lotus (Nelumbo nucifera Gaertn)promote root formation and enhance stress tolerance in transgenic Arabidopsis thaliana
Source: BMC Genomics. 2023 Nov 28;24:719. doi: 10.1186/s12864-023-09772-w (PMC10683310; doi:10.1186/s12864-023-09772-w)
Supplement: Supplementary file 1 — Additional file 1: Table S1. [file 12864_2023_9772_MOESM1_ESM.docx]

Additional table.1. Gene sequences of *NnWOX1-1*, *NnWOX4-3* and *NnWOX5-1*.

***Nnwox1-1*:**

ATGTGGATGATGGGTTGCAGTGATGGAAGTGGGTTTAACATGGCGGATTCTTTCAACGGTCGGAAGCTTCGGCCTCTCATGCCAAGGCTTACTACTAATAGCACTAACACTGCAACTGTAATGGCTCCTTGCTTGACTCGTATTCATGGTACAGATTTCTTTGCGTTGAATACTCATCTGGCCACTGTAACCGAACACAGTAAGAGAGAGTTCAGTGCACAACCAGTGGTGAGCTCCCGGTGGAACCCCACACCGGAGCAGCTACGGACCCTCGAGGAATTATATCGATGTGGAACTCGAACGCCGACGGCTGAGCAAATCCAGCACATCACTGCTCAGCTCCGCCGGTTTGGCAAGATCGAAGGGAAGAATGTGTTCTATTGGTTTCAAAACCACAAGGCAAGAGAAAGGCAGAAACGCCGTCGTCGTTCTGCTGCTTCCGAAGAACAACAGCACTACGATACTGAAAGCTTAGACAAGAAAGAATCAGGGTCGAGTAGGACAGGCTATGAAGTTGAACAGACCAAGAACTGGGCTCCCCCTACAAACTGCAGTAGCCTTTCAGAGGAAACTGTTTCAATGCAAAGAGCAGCAGTAGCAGAAAGTAGAACAGACGGGTGGATTCAATTCGAGGAGGGAGAATTACAGCACAGAAGAACCTCAGTAGAAAGGAATCCTACGTGGCAAATGATGCAGCTATCTTCTCCTCCCACCACCCCTCTCAAAAACACCATAACCACAGCAACAACAGAAGCAATAACAGTAGTAGACCCAAAGCTCATAAACAACCAAAATCTGGAGCTTTTTAAAACACCCAGCAGAGAATATCTCCCAGCTGATCTCCTCAGCAATGGCGAAGAACGCAAAGACCAGGAGGAGTGTGGAGAATCTCAAACGCTTCAGCTCTTCCCACTAAGAAGCGACAGTGGAAATGGTGGTGTCGGTGATGAAGAGAAGGAGACTGAGGTGCCGAATCCAACCACGAATTCCAACTTTACTACATACCAATTTTTTGAGTTCCTTCCCACGAAGAACTGA

# *Nnwox4-3:*

ATGAGAAGCTTGAACATGAAGGTTCATCAGCTTGCACGTGGGCTATGGGAGCATGAACCCTCCCTCACACTTGGCTGCAAGCGCCTAAGGCCTCTTGCACCCAAGCTTACTAATGGTGAAACTCCGGCTATTCTTGACCTCAAAAGCTTCATCAAGCCTGAAAGTAGTAGTGGACCTAGCAAGTCCGACGACAAGCGAGAAGCCGTTCAGGTGGACGCTCCTTCTGGAGGCACAAGGTGGAACCCTACACAGGAGCAGATAGGAATCCTAGAGATGCTCTACCGTGGTGGAATGCGTACCCCCAACGCCCAACAGATAGAGAAAATCACTGCTCAGCTTGGCAAGTTTGGGAAGATTGAAGGAAAGAACGTTTTCTATTGGTTCCAAAACCACAAAGCGCGGGAGAGACAGAAGCAGAAGCGCAACAGTTTGGGTCTTGTTCATTCTCCAAGGACTCCTCCCGCTACCACTGTAGCCTTGGATGTTAATAGGGACGAAATAGAAAGAGAAGAAGATAGTCACTACAAACGGAAACACCGAGGCTGGGCAATCGAATTCTTCCAACAGGATAGAGGGTTTTGTGGAGGAGGAGAAGAGGGAGATAAGACACTGGAACTCTTCCCTTTGCATCCGGAAGGCAGATGA

***Nnwox5-1:***

ATGGATGAACGAATGTTGGGTTTCTGTATTAGAGCTGGAGGATATGGATATGGTGGTGGTGGTTGTCATGCAGCTGGAGGAGGTGGTGGAGGAACCAAGTGCGGGCGTTGGAATCCGACAGCAGAACAAGTTAAAGTTCTCACTGATCTTTTCAAATCCGGGCTCCGAACTCCGAGCACTGATCAGATCCAGAAGATCTCAACTCAGCTCAGCTTTTACGGAAAGATAGAGAGCAAGAACGTCTTCTACTGGTTTCAGAACCATAAAGCCAGAGAAAGGCAGAAACGCAAGAGAGTATCTGTCGACGACCAAGATTTCATCGACAGAGCAGTGGACAAAGTTTTACCAGCAACAAAACATTTCGTGGAAGTTGATCTCGTTTCGGAGCCTGAAAGAACCATAGAAACGCTGCAGCTCTTTCCGCTGAACTCGTACCAGGATTCGGACTCCCTGAAGCTGAGACTATTTAGGAACGAATACAAGGAAACGACATTCTCGTATGGGATCGGGAAAGAGAATGATCATCCACCATTGGATCTTCGTTTAAGCTTTCTGTGA
