# Supplementary figures and images for: NnWOX1-1, NnWOX4-3, and NnWOX5-1 of lotus (Nelumbo nucifera Gaertn)promote root formation and enhance stress tolerance in transgenic Arabidopsis thaliana
Source: BMC Genomics. 2023 Nov 28;24:719. doi: 10.1186/s12864-023-09772-w (PMC10683310; doi:10.1186/s12864-023-09772-w)

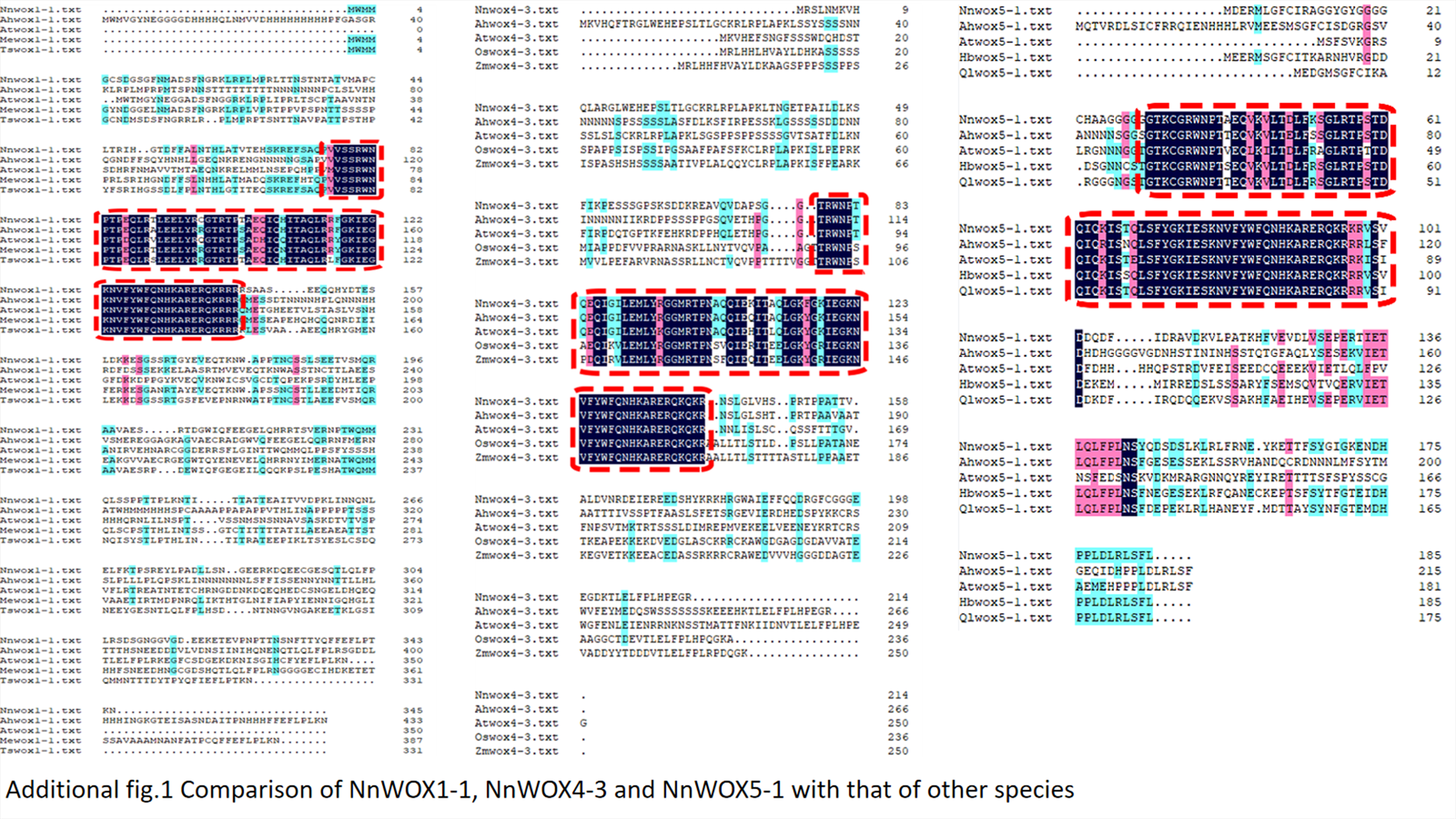

Supplement: Supplementary file 2 — Additional file 2: Fig. 1. [file 12864_2023_9772_MOESM2_ESM.tif]

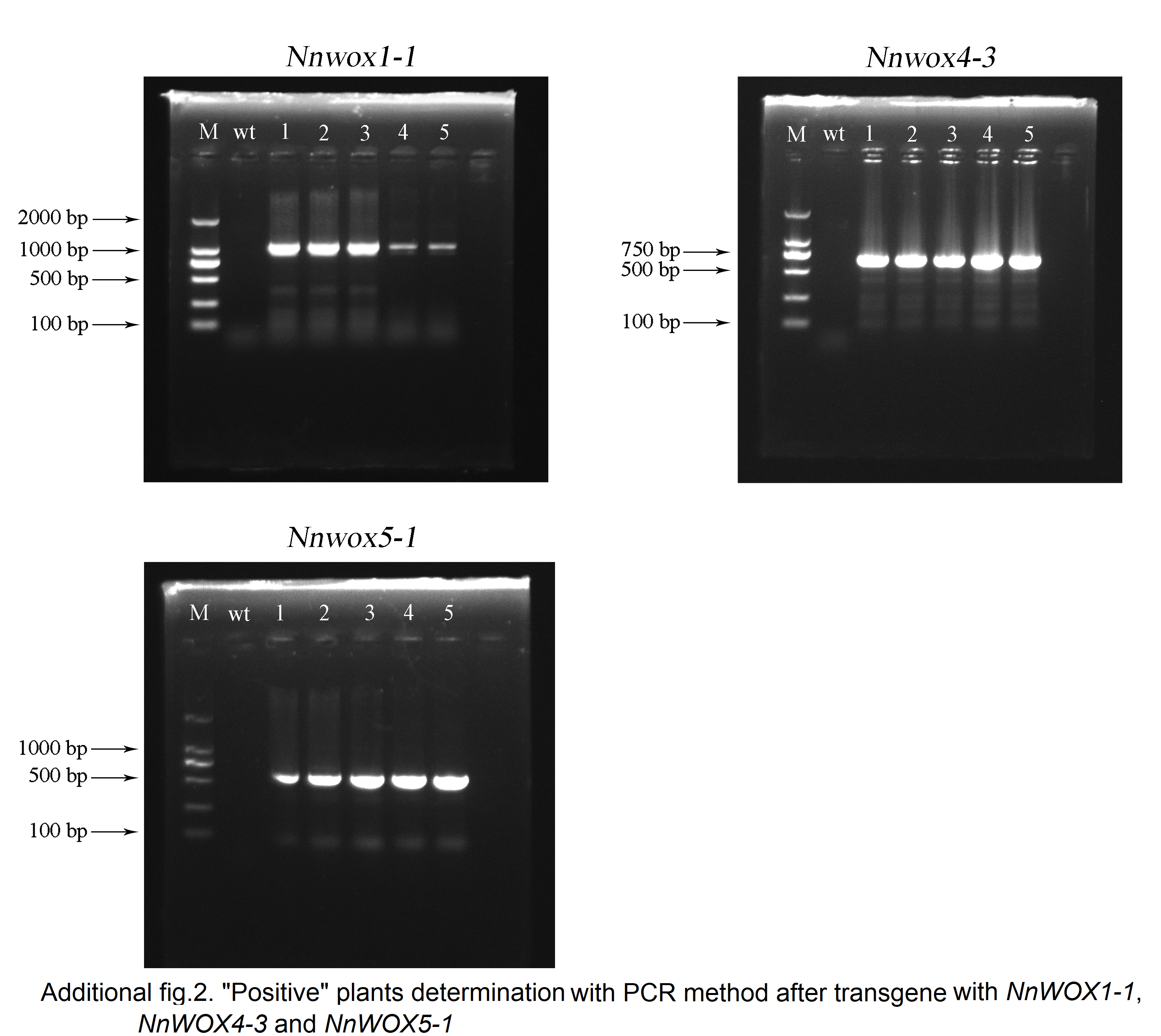

Supplement: Supplementary file 3 — Additional file 3: Fig. 2. [file 12864_2023_9772_MOESM3_ESM.tif]

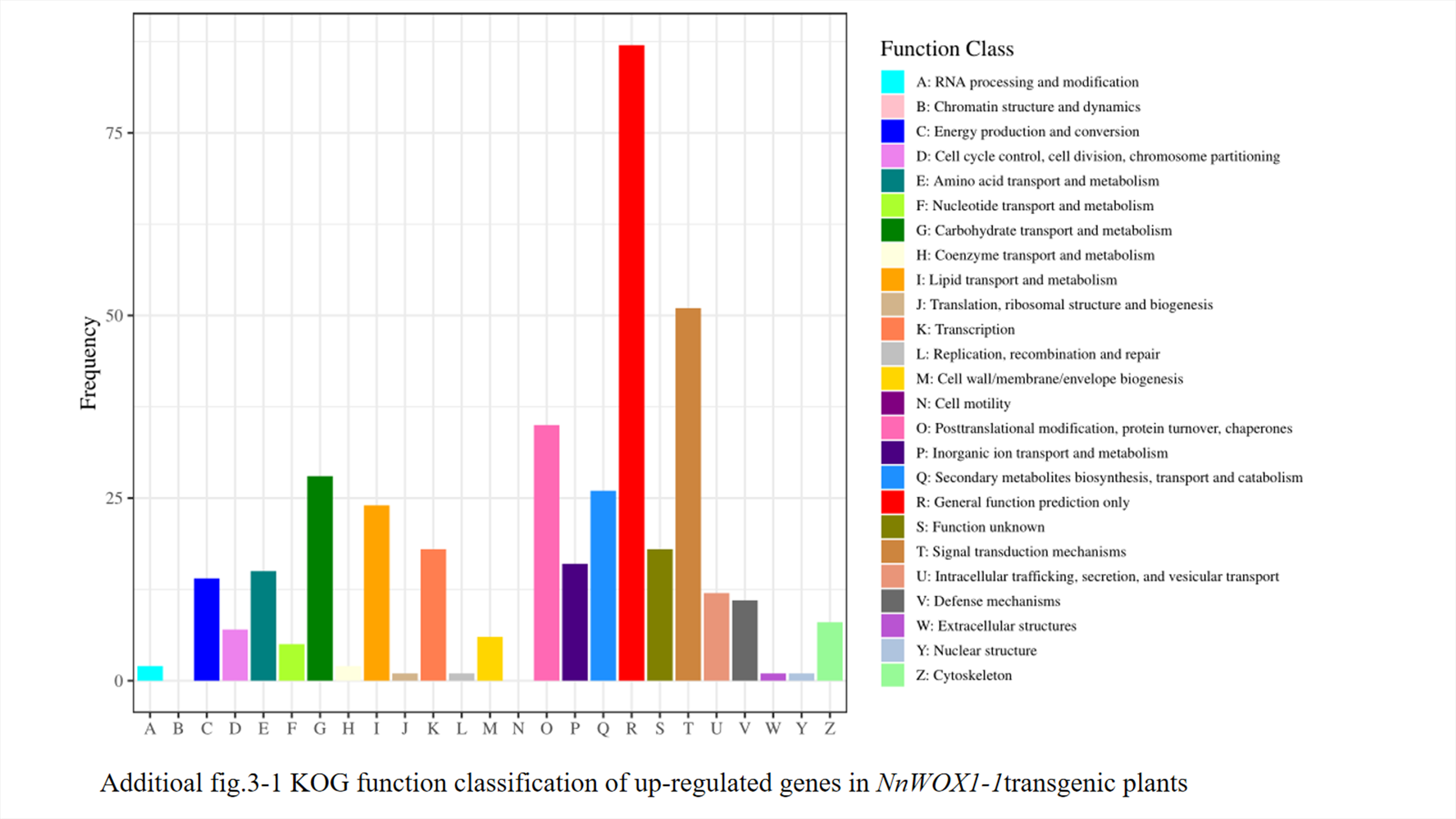

Supplement: Supplementary file 5 — Additional file 5: Fig. 3. [file 12864_2023_9772_MOESM5_ESM.zip › Additional fig. 3-1.tif]

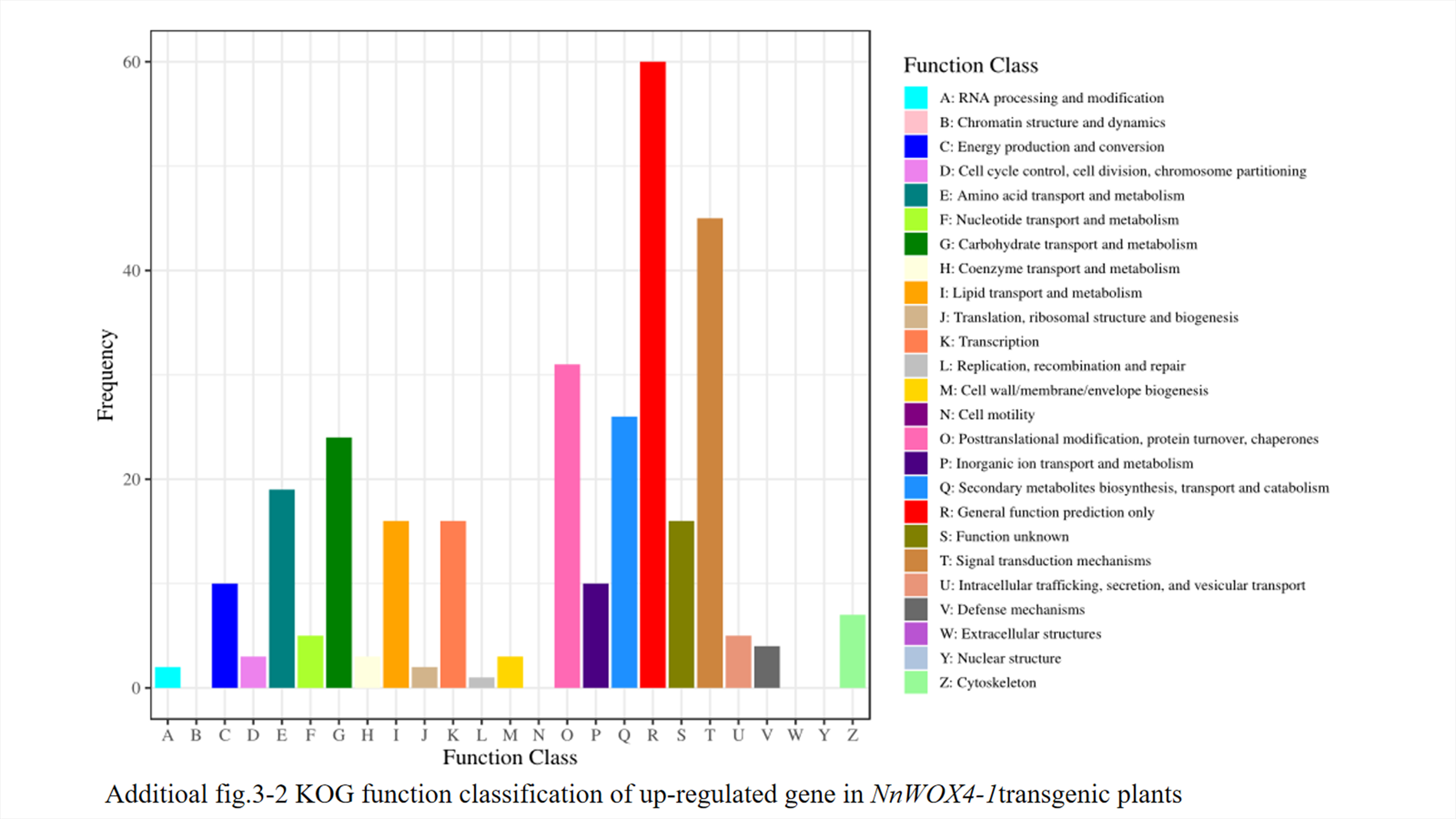

Supplement: Supplementary file 5 — Additional file 5: Fig. 3. [file 12864_2023_9772_MOESM5_ESM.zip › Additional fig.3-2.tif]

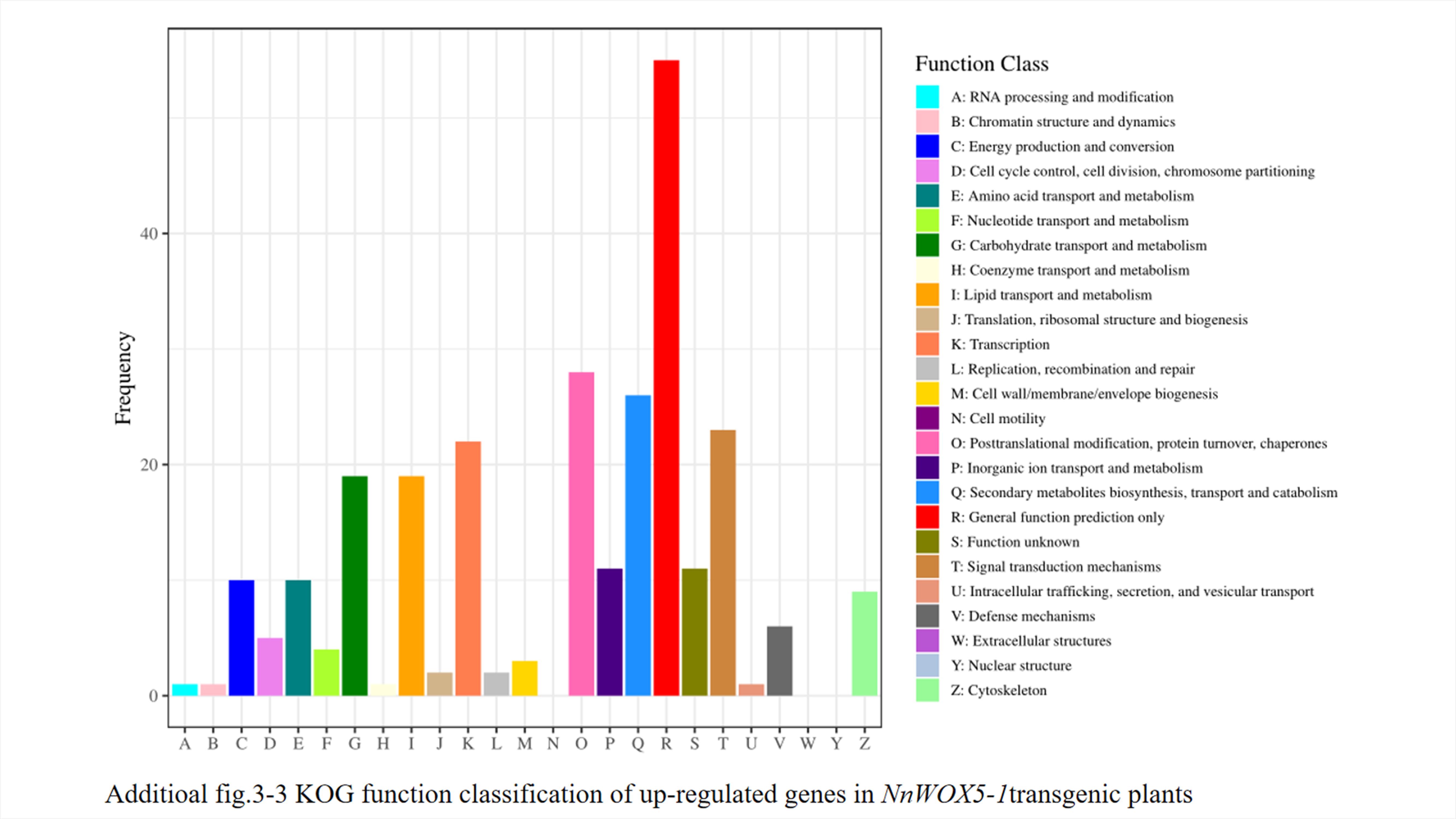

Supplement: Supplementary file 5 — Additional file 5: Fig. 3. [file 12864_2023_9772_MOESM5_ESM.zip › Additional fig.3-3..tif]
